# Do Co-Worker Networks Increase or Decrease Productivity Differences?

**DOI:** 10.3390/e23111451

**Published:** 2021-10-31

**Authors:** László Lőrincz

**Affiliations:** 1 NETI Lab, Corvinus Institute for Advanced Studies, Corvinus University of Budapest, 1093 Budapest, Hungary; laszlo.lorincz@uni-corvinus.hu; 2 ANet Lab, Institute of Economics, Centre for Economic and Regional Studies, 1097 Budapest, Hungary

**Keywords:** labor mobility, co-worker networks, regional inequality, knowledge spillovers, agent-based simulation

## Abstract

Do labor mobility and co-worker networks contribute to convergence or divergence between regions? Based on the previous literature, labor mobility contributes to knowledge transfer between firms. Therefore, mobility may contribute to decreasing productivity differences, while limited mobility sustains higher differences. The effect of co-worker networks, however, can be two-fold in this process; they transmit information about potential jobs, which may enhance the mobility of workers—even between regions—and this enhanced mobility may contribute to levelling of differences. However, if mobility between regions involves movement costs, co-worker networks may concentrate locally—possibly contributing to the persistence of regional differences. In this paper, we build an agent-based model of labor mobility across firms and regions with knowledge spillovers that reflects key empirical observations on labor markets. We analyze the impact of network information provided about potential employers in this model and find that it contributes to increasing inter-regional mobility, and subsequently, to decreasing regional differences. We also find that both the density of coworker networks, as well as their regional concentrations, decrease if network information is available.

## 1. Introduction

Worker mobility is a major source of transferring knowledge between firms, as firms utilize the incoming personnel’s knowledge and skills that they have acquired throughout their careers [1,2,3,4]. A piece of direct evidence for this knowledge spillover is that hiring workers from better-performing firms increases the recipient firms’ productivity [5,6,7,8]. In addition, increased wages of workers at the recipient firm after hiring personnel from high-performing competitors indicates the within-firm diffusion of new knowledge [9].

These knowledge spillovers through labor mobility have implications for productivity differences within sectors or regions as well. Knowledge transfers between firms may decrease productivity differences, while constraints to knowledge transfer can explain why productivity differences are sustained. An example of sustained productivity differences has been observed in the U.S. manufacturing sectors, where the productivity of the 90th percentile is twice the productivity of the 10th percentile on average, and even higher in some sectors [10]. In India and China, even higher differences have been observed [11]. Previous studies have concentrated on the (lack of) market competition when explaining these differences [12,13], or competition advantages due to export activities [14]. However, the role of labor mobility was not examined in these studies.

Regarding regional analysis, high levels of mobility between related industries, and an increasing density of co-worker networks are shown to contribute to higher growth rates of regions [15,16,17]. Different mechanisms were proposed to explain this finding. First, high mobility can contribute to agglomeration externalities. Second, dense coworker networks also induce better employer–employee matching. In addition, network density can be an indicator of social capital and trust, which supports learning from contacts [15,16,17].

Furthermore, knowledge transfer via labor mobility may also contribute to the catch-up of lagging regions to more developed ones. Skills of migrants returning from more developed regions may boost the productivity of a local sector [18,19], which has been shown when external shocks—such as the economic crisis—or changes in immigration policy have forced many migrants to return home.

Why, however, would migrants move (back) to lower-developed regions in the absence of such shocks? Unrealized expectations in returns for skills (e.g., low wages and unemployment) may be one reason [20], but potential returns of accumulated human capital together with lower price levels may be another [21,22].

Our first aim, therefore, is to connect these studies by creating a model of voluntary labor mobility with which we can assess how labor mobility levels up within- and between-regional productivity differences, and how obstacles to labor mobility contribute to preserving these differences. Our second aim is to examine the role of co-worker networks. Although we have empirical observations about regional growth and co-worker networks [15,16,17], we know less about the mechanisms, i.e., *how* they contribute to the catching-up of regions. Furthermore, while the role of obstacles to labor mobility in sustaining regional differences is relatively straightforward to predict, the role of co-worker networks in this picture is less simple.

Not only do networks of former coworkers serve as transmitters of knowledge between firms, they also convey information about employees and employers. As the labor market is characterized by imperfect or asymmetric information, this influences labor mobility in different ways [23]. First, networks may transmit information about job vacancies to unemployed persons. This predicts that employment probability is correlated across social networks, and that network size increases the chance of employment [24]. In this regard, it has also been shown that an increased employment rate across former coworkers strongly increases workers’ re-employment probability after unemployment [25].

Secondly, information available from former coworkers decreases the uncertainty of employers about the “quality” of candidates [26]. This model shows that the consequence of having former co-workers at a company is increased starting wages. The existence of such a wage gain has been shown empirically—a fact that has been explained by two rationales: First, that by network information firms can select workers with better unobserved skills, and secondly, that such networks enable workers to choose from higher productivity (and thus higher paying) firms [27,28]. Another consequence is that employers are more likely to hire workers with whom their current workers have connections [29].

A third approach assumes that workers’ networks transmit information about the employer–employee fit [30,31,32]. They assume, based on the matching model of Jovanovic [33], that each worker has a potential (productivity) that is firm-specific. That is, different workplaces require workers with different skills, and if they match, that makes the worker productive. However, being successful at one firm does not necessarily mean that the same worker will be successful at a different one. This matching factor is assumed to be unknown to the workers and firms a priori, and is revealed to them over time with employment, or by network information. Supporting empirical evidence of this model includes the fact that referred workers have higher initial wages and lower turnover than non-referred ones, and that this wage difference gradually declines with tenure [30,32]. A further consequence is that information on matching makes employers more attractive where former coworkers are present; thus, there is a tendency for workers to follow each other across firms [32].

Concerning the regional impacts of this, job referrals especially facilitate job transitions between different regions, e.g., the movement of workers from rural areas to the city [34]. Therefore, with more extended coworker information networks, increased mobility between regions can be expected at the first instance. This increased mobility, due to knowledge spillovers, may therefore be expected to decrease regional differences. Relatedly, concerning the impact of networks on geographical mobility, it is known that social networks between regions create self-sustaining migration systems [35], which suggests that the initial connections may cause persistent effects.

However, it is a widely observed property of geographic mobility that it is negatively related to distance, as mobility over long distances includes different material and non-material costs, e.g., [36]. This implies that coworker networks also tend to cluster locally [37]. People with more extended local networks, furthermore, tend to be less likely to move [38,39]. It is therefore also possible that the more extensive the network information, the greater the tendency of forming local concentrations of coworker networks; thus, coworker networks may not contribute to decreasing regional differences at all, or may even amplify them.

Accordingly, we examine a model of labor mobility and productivity spillovers by adding the informative role of co-worker networks. Utilizing this, we study the relationship between mobility and productivity differences within and between regions, and the specific role of co-worker information in this relationship.

## 2. Method

An analytical model of voluntary labor mobility with heterogeneous workers and firms is in itself a rather complicated exercise (a well-known example is by Burdett and Mortensen [40]), and there are also useful examples for modelling labor mobility together with network information, e.g., [31]. We believe, however, that applying an analytical model of voluntary labor mobility to heterogeneous workers and firms with network information and productivity spillovers would be extremely difficult. Therefore, to study the relationship between these phenomena, we turn to the technique of agent-based modelling. Agent-based models originate from equation-based models in natural sciences, which are widely applicable to problems in socio-economic sciences [41]. They assume independent, adaptive, and autonomous actors that follow simple rules, which is congruent with the foundations of economics and micro-sociology. The key assets of the models that we utilize are that they can serve as experiments for social sciences, and for studying complex, emergent outcomes of systems that are not directly derivable from individual actions [42], or from what one could derive from a mean-field mathematical model. For our purpose of studying labor mobility, these are important features, as real experiments are constrained by ethical considerations—and even the possibility of empirical analysis is limited to partial relationships in which external shocks can be utilized due to the endogenous relationships between our variables (e.g., between mobility and productivity differences).

When creating the model, we built on general assumptions of existing models in labor economics to maintain comparability, and took into consideration the generic nature of our assumptions. Empirically, we set parameters according to existing studies where observations were available, and tested our predictions on different parameter settings, considering those parameters where no such observations existed. We used the *Netlogo* program for the simulations. The code for the simulations is included in the Supplementary Materials.

### 2.1. Initial Setting

To start, we initially distributed the $N_{W}$ workers across the $N_{F}$ firms. We generated an initial heterogeneity in the firms’ productivity according to uniform distribution: *A = {U 0,1}.* This represents the heterogeneity of the capabilities of firms, which may be associated with “managerial talent”, corresponding to the model of Lucas [43].

We assumed that a fixed, β share of firms’ productivity was paid to the worker as a wage; thus, the wage of each worker at firm *j* was $W=\beta A_{j}$. In addition, each worker had a firm-specific non-wage utility,$\;\mu_{ij}$ which was also drawn from a uniform distribution, $\mu_{ij}$ = *{U −0.5*,*0.5}*. This added the heterogeneity of workers’ preferences to the model. This represents how much workers like the type of the job they do at the firm, across dimensions in which taste is heterogeneous; for example, whether they have to be autonomous, or have well-structured tasks; if they have to work in teams or independently; if it requires social or analytic skills, etc. Workers consider this non-wage utility together with wage, and thus, they maximize their total utility, $W_{j}+\mu_{ij}$.

An important property of this setting is that workers were homogeneous in the sense that they did not differ in their productivity (skills). In this sense, our approach is different from Lise, Meghir, and Robin [44] and Lopes de Melo [45], and similar to Pissarides [46] and Nagypál [47]. Our approach was also related to the setting of Simon and Warner [30], Dustmann et al. [31] and Glitz and Vejlin [32], in the sense that we assumed that the match between firms and workers was firm-specific; however, in those models, workers have heterogeneous productivity (which is firm-specific)—while in our study, the match-specific component was the non-wage utility. Furthermore, in those models, the firms are homogeneous—while in our case, the productivity of workers was similar. The reason for our choice of analyzing workers with similar skills was that having heterogeneous workers together with heterogeneous firms would raise the question of a positive interaction between workers’ productivity and firms’ productivity, and the subsequent sorting of high-ability workers to high-productivity firms [48]. In addition, this setting would call for the analysis of another role of social networks—the signaling of worker productivity to firms [26]. Thus, although the influence of sorting on mobility—and the role of co-worker networks in this process—is an important question, we believe that it would be more appropriate to analyze that question in a different model.

Concerning parameter β, we can interpret it as the bargaining power of the workers, similar to the model of Nagypál [47]. For our purposes, however, it was exogenous and constant, and not specific to the worker–firm relationship. In specific terms, we can manipulate the weight of the non-wage utility compared to wage by this parameter. We set this parameter to β=0.5, but also tested how it influenced the equilibrium.

Our workers represent overlapping generations; they were active for forty periods—thus, one period corresponded to one year on the labor market. Afterwards, they retired, and new workers replaced them. We started the model with heterogeneous initial experience to avoid periodic fluctuations.

In the model, we assumed imperfect information, where workers are uncertain about their firm-specific fit (non-wage utility, $\mu_{ij}$) at their prospective workplaces. They choose, therefore, based on $W_{j}+E(\mu_{ij})$, where $E(\mu_{ij})$ is the average value in the population. However, once employed, the true $\mu_{ij}$ parameter for their current employment is revealed to them. At a later stage, we introduced the network information that reveals the true $\mu_{ij}$ parameter for the potential workers. Thus, in this respect we followed the network information models of Simon and Warner [30] and Dustmann et al. [31].

Similarly, we introduced labor mobility by assuming that jobs were destroyed at an exogenous rate, δ. Workers whose jobs were destroyed had to choose a new job (N.B. the model would be very similar if we did not assume job destruction, but rather that people at different points of their life change their preferences about jobs). In addition, workers with existing jobs may also change workplaces. Both groups are allowed to choose among the available offers, which arrived at a rate λ. We set the job destruction parameter to δ=0.1, as it directly influences the mobility rate, and we would like these parameters to be around the empirically observable range (job separation and hire rates have been 8–16% in the U.S in recent decades [49]). The parameter of the arrival rate allowed the model to reflect real-world decisions better compared to the perfect information assumption. We set the arrival rate parameter at λ=0.1 levels throughout the simulations and tested how it influenced the equilibrium.

### 2.2. Labor Mobility and Knowledge Spillovers

The special feature of our model is that we added knowledge transfers to the labor mobility model. We assumed that if workers move, the new firm may utilize some of their experience, creating a productivity spillover. We specified this the following way: The movement of a worker from firm a to firm b yields:

(1) $$\begin{array}{c} \left\{\begin{array}{c} {A^{\prime }}_{b}=A_{b}+\frac{\left(A_{a}-A_{b}\right)\theta }{N_{b}}\;if\;A_{a}>A_{b}\;\\ {A^{\prime }}_{b}=A_{b}\;if\;A_{a}\leq A_{b}, \end{array}\right. \end{array}\;and\;{A^{\prime }}_{a}=A_{a},$$

where $A^{\prime }$ is the changed productivity parameter, θ<1 is a parameter representing the transferability of knowledge, and $N_{b}$ is the number of workers at firm b. This specification is identical to Stoyanov and Zubanov [6] and Csáfordi et al. [8] in the formulation that the weight of new knowledge brought by a single worker decreases by the number of incumbent workers, and corresponds to their empirical findings that negative productivity differences do not cause changes. We set the spillover parameter to θ=0.3, corresponding to the empirical estimates in these studies.

Turning back to the decision on mobility, we assumed that this productivity spillover was incorporated into the wage offer, so firms offer wages to workers based on their previous careers and their subsequent future productivity. We also assumed that the mobility of workers is costly, and therefore, that workers leave their workplace only if their benefit exceeds a switching cost parameter SC. Therefore, the worker i switches from firm a to firm b if:

(2) $$\left\{\begin{array}{c} E\left(\mu_{ib}\right)+\beta \left(A_{b}+\frac{\left(A_{a}-A_{b}\right)\theta }{N_{b}}\right)>\mu_{ia}+\beta A_{a}+SC\;if\;A_{a}>A_{b}\;\\ E\left(\mu_{ib}\right)+\beta A_{b}>\mu_{ia}+\beta A_{a}+SC\;if\;A_{a}\leq A_{b} \end{array}\right.$$

If the productivity of a recipient firm increased due to the experience of the incoming workers, we assumed that this firm also increased the wages of its incumbent worker accordingly, from $W=\beta A_{j}$ to $W^{\prime }=\beta {A^{\prime }}_{j}$, so that there would be no wage differentials within the firm between the workers (who were assumed to be similar in skills). This positive externality of new knowledge on the wage of incumbent workers was in line with the results of Poole [9].

We implemented labor mobility in two steps: First, those workers whose jobs were lost look for a job; they choose from the available offers according to Equation (2). As the subsequent productivity spillovers influence the optimal choice of those workers whose jobs were not lost too, we next re-considered their workplace choice, allowing voluntary mobility. The number of offers workers can choose from was determined by the arrival rate parameter, so they get $\lambda N_{F}$ offers. Next, they consider these alternatives and consider the best one according to the left-hand side of Equation (2). If they find this best alternative favorable enough, that is, if the inequality in Equation (2) holds, they take this option—otherwise, they stay. In the model, the extent of mobility is influenced by the extent of job loss and the switching cost parameters, of which the first remains fixed, and only the switching costs are manipulated. To sum up, each turn of the simulation consists of the following subsequent steps:

1. Jobs are lost, and the workers affected look for a new workplace.
2. The mobility of these workers creates productivity spillovers that may change the productivity of firms, and firms update the wage of their workers according to the new productivity levels.
3. Workers are given the opportunity of voluntary mobility.

This setting neatly reproduces the key empirical observation in labor economics that larger firms offer higher wages [50]. This correlation in the model follows from the assumption that firms are heterogeneous in their capabilities (i.e., productivity), and that more productive firms pay higher wages—therefore, they are more likely to attract more workers, as suggested by Lucas (1978). However, in our setting, it was not the decreasing marginal returns in the production function, but rather the heterogeneity in workers’ non-wage utility that prevented the firm with the highest capability from taking over the whole labor market.

### 2.3. Regions

Our key interest in this study was the effect of labor mobility on regional differences. To study this, we introduced regions, trying to keep the model as simple as possible. To be able to assess whether labor mobility contributes to the convergence of the regions, we created two regions with different average productivity levels. At the beginning of the simulation, firms with equal probability were allocated to regions at random. Next, the initial productivity of firms was determined randomly, and a constant “regional difference” parameter was deducted from the firm if it is in Region 1. Thus, one can also think of Region 2 as being in a more developed center, and Region 1 as a representative for the less developed periphery. We did not allow firms to relocate between regions, but workers could move between them.

However, we assumed that mobility for workers is more costly between regions, than moving to a firm within their current region. Accordingly, we assumed that when changing jobs, they face two types of mobility costs: they bear a general switching cost (*SC*) if they change workplaces, but if they choose a firm in the other region, the cost of moving (*MC*) adds to this. This modified the condition under which worker i moves from a more productive firm a to a less productive firm b the following way:

(3) $$\;E\left(\mu_{ib}\right)+\beta \left(A_{b}+\frac{\left(A_{a}-A_{b}\right)\theta }{N_{b}}\right)>\mu_{ia}+\beta A_{a}+SC+MC$$

where *MC* = 0 if firm a and b are located in the same region.

### 2.4. Innovation

In this setting, the following dynamics can be observed. If a worker moves from a less productive firm to a more productive one, the productivity differences between them do not change. However, if a worker moves from a productive firm to a less productive one, the difference in productivities decreases. Therefore, productivity differences will continuously decrease, unless there is no mobility in the system. However, in reality, we experience persistent mobility *and* productivity differences, so it would be a more appropriate property of the model to reflect this phenomenon. Therefore, we had to introduce a force of divergence of productivities to the model—which will be innovation.

We borrowed this idea from the “escape competition” model of Aghion and Griffith [51], and assumed that more productive firms are more likely to develop a productivity-enhancing innovation (however, we assume that others do not imitate the innovators, but learn through labor mobility).

In our system, in each round *one* firm innovates, which is costless, and increases its productivity by a constant parameter of INN. The selection of the innovating firm is random; however, it is not made with uniform probability—the likelihood of each firm becoming the successful innovator is proportional to its current productivity, thus:

(4) $$pr\left(innovator=a\right)=\frac{A_{a}}{\sum_{j}A_{j}}.$$

In the simulation, innovation thus consists of two steps:

1. Selecting the one innovator firm according to Equation (4).
2. Adding *INN* to its current productivity.

We implemented innovation directly before the step of voluntary labor mobility, so workers could adjust to this by voluntary mobility. Furthermore, as a final adjustment, we deflated the productivities by the change in the average productivity of all firms at the end of each round. This is necessary because otherwise, productivity would grow continuously in the system, which would result in higher and higher wages; thereby, the weight of the non-wage element in the workers’ choice would slowly but gradually vanish.

## 3. Results

### 3.1. Equilibrium in the Basic Model

**Figure 1:**
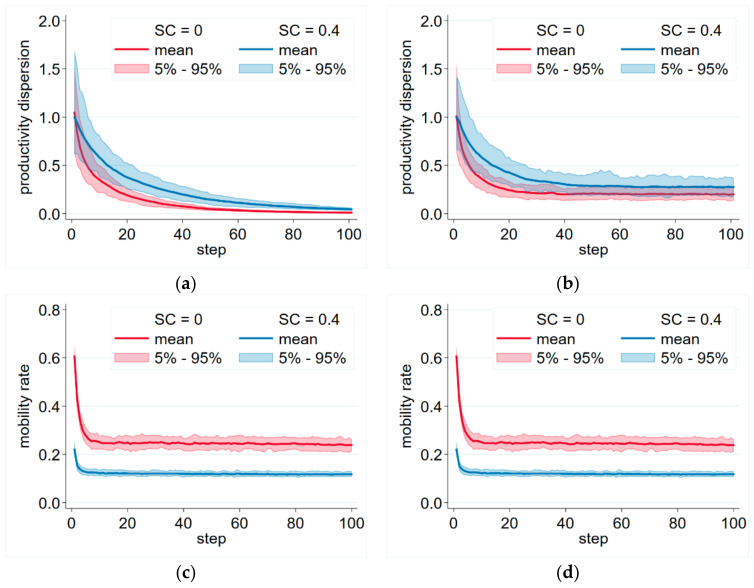
Productivity dispersion and mobility with and without innovation. (**a**) Productivity dispersion with no innovation (*INN* = 0). (**b**) Productivity dispersion with positive innovation (*INN* = 0.1). (**c**) Mobility with no innovation (*INN* = 0). (**d**) Mobility with positive innovation (*INN* = 0.1). Productivity dispersion is measured by the interquartile range divided by the median, following [10]. Parameters: $N_{p}=300\;persons,\;N_{f}=30\;firms,\;\beta =0.5,\;\delta =0.1,\;\lambda =0.1,\;\theta =0.3$.

After including innovation in the model, it produced persistent mobility and productivity dispersion over a reasonable set of parameters. After an initial adjustment, the simulations stabilized on an equilibrium level of mobility and productivity dispersion (Figure 1b,d). It was also observable that if we increased switching costs, this equilibrium level of productivity dispersion decreased and the level of mobility increased. On the other hand, without innovation, the productivities of the firms converged and the productivity dispersion disappeared, even if switching costs were present (Figure 1a).

Intuitively, the presence of a stable level of productivity dispersion originates from the following two factors: First, if a firm gets more productive, it attracts more workers and grows bigger, at the expense of the others, which become smaller. However, these small firms benefit more if they can gain a worker from the more productive one, as the mobile worker’s knowledge disperses more easily in a small community. This is represented by the number of the recipient firm’s workers ($N_{b}$) in the denominator of the spillover formula in Equation (1).

Additionally, as the firm grows bigger, the chances increase that a randomly selected worker whose job has been lost will be drawn from that firm.

**Figure 2:**
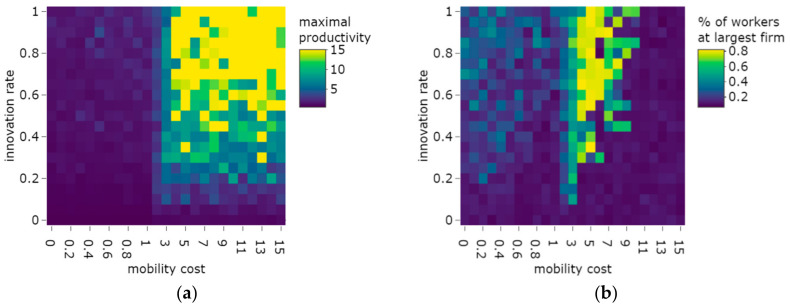

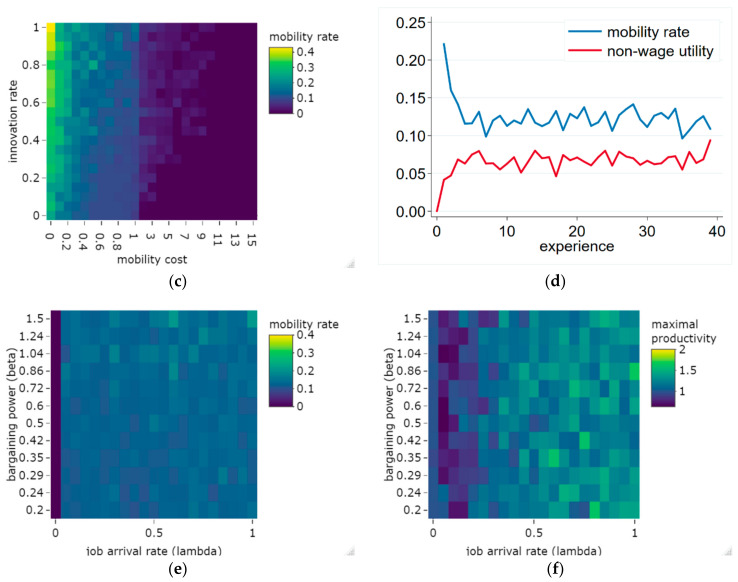
Equilibria over different ranges of the parameters. (**a**) The effect of the mobility cost and innovation rate on maximal productivity. (**b**) The effect of the mobility cost and innovation rate on the largest firm’s size. (**c**) The effect of the mobility cost and innovation rate on yearly mobility rate. (**d**) Mobility and non-wage utility by workers’ experience. (**e**) The effect of the job arrival rate and bargaining power on mobility. (**f**) Maximal productivity by job arrival rate and bargaining power. Notes. (**a**–**c**): Each dot represents one simulation at the 1000th step (a higher number of steps was necessary to study the equilibria due to the inclusion of extreme values). (**d**): Each line represents the average of 10 simulations at the 100-th step. (**e**,**f**): Each dot represents one simulation at the 100th step Parameters: $N_{p}=300\;persons,\;N_{f}=30\;firms,\;\beta =0.5,\;\delta =0.1$. (**a**–**d**): λ=0.1, θ=0.3.

One can expect, however, that if mobility costs increase over a certain threshold, some firms can gain some productivity by the innovation mechanism, and consequently, pay such high wages that moving away to any other firms will not be attractive for any of its workers, because the productivity spillovers and the difference in non-wage utility cannot compensate the wage differences any more—thereby it slowly overtakes the whole labor market. In this case, they may also benefit from the cumulative nature of the innovation mechanism (that innovation happens with a higher probability at firms that already have high productivity), and increase their productivity further and further away from the other firms; thus, they successfully “escape” from the competition. The emergence of this phenomenon is examined in Figure 2, with concern to mobility costs and innovation rates. Figure 2a suggests that within the reasonable range of parameters ($SC\in \left[0,1\right],\;INN\;\in \;\left[0,1\right])$, firms are not able to escape from their competitors, as labor mobility transfers the innovation to competitors. Still, if the innovation rate increases, the highest productivity firm tends to have some advance in productivity. If the innovation rate is high and the cost of mobility is low, these firms gain a significant share of workers; however, they do not overtake the whole market (Figure 2b). Firms escaping the competition by innovation only happens if the mobility costs are increased to a very high level (SC>3), and if the innovation rate is also high. The yellow area in Figure 2a shows that one firm escaped from the competition (As the mean productivity in the beginning is 0.5, and the average productivity over the simulations is normalized to this level, the highest productivity value of 15 indicates that one firm of the 30 ones has productivity 15, and all others have 0). In the range of switching costs of 3<SC<8.5, these firms tend to take over the whole labor market, which happened in the following way: At the beginning of the simulation, productivity levels are relatively even, and very high switching costs prohibit any labor mobility. Due to the high innovation rates, a ‘fortunate’ firm tends to increase its productivity and start to gain a significant advantage. After getting such a high productivity advantage that the wage difference exceeds the switching cost, workers from the lagging firms can move to the high productivity firm, but the switching costs prohibit mobility in the reverse direction. In some of the simulations, it is not one, but two firms that escape from the competition, and a duopoly emerges—indicated by the green dots on Figure 2a,b. If, however, the switching costs are increased to an even higher level (*SC* > 8.5 in the 30 firm setting), they tend to prohibit all mobility—even between a firm with a maximum and minimum productivity level. Therefore, a firm typically escapes the competition by innovation, but it cannot overtake the labor market, as mobility is zero (Figure 2c).

It can be observed that innovation adds additional motivation for mobility. If a firm innovates, its productivity increases together with the wages offered by it, which creates the motivation to join the firm, which in turn opens up the opportunity for new mobility. Therefore, the combination of maintaining low switching costs and raising the innovation rate increased mobility (Figure 2c). Taken together, the analysis indicates that the dynamics of the model were stable over a wide range of the parameters ($SC\in \left[0,1\right],\;INN\;\in \;\left[0,1\right]$); as such, our analysis did not concentrate on an extreme setting.

Examination of the workers over their life cycle reveals that their mobility rate was the highest at the beginning of their career, when their firm-specific non-wage utility increased. Later they found their ideal jobs, and their non-wage utility stabilized, and mobility settled at a lower level (Figure 2d). This corresponds to the empirical observations of the labor economics literature [52].

Concerning the impact of the bargaining power and job arrival rate parameters, mobility rate was hardly affected by these (Figure 2e); except in trivial cases, i.e., if the job arrival rate was zero (workers have offers to choose from), mobility was consequently zero. A small positive impact of the beta parameter could be observed, which was due to the increased available wages (as wage is productivity multiplied by beta) compared to the fixed switching costs. Productivity differences, however, were influenced more by the job arrival rate (Figure 2f). In cases where the job arrival rate was low, mobility contributed to leveling up productivity differences compared to when there was no mobility (λ=0). On the contrary, when the arrival rate was high, i.e., when mobile workers were allowed to get admitted to any firms on the market that they wished, productivity differences increased. In this case, workers could pick the highest productivity (best-paying) firms, so high-productivity firms would employ the bulk of the workers, who would not move to lower-productivity firms; thus, knowledge transfer would be limited.

### 3.2. The Effect of Network Information

We examined the effect of co-worker networks by adding the following assumptions:

1. Workers have no initial information about their non-wage utility parameter at prospective employers if none of their former co-workers works there, but
2. if they have a former co-worker at a firm, their true parameter is revealed for them initially.

This information may influence mobility both positively and negatively. Information on a bad personal match may dissuade workers from moving to a specific firm, but information on a good match may encourage mobility.

To get an idea about the effect of this information, consider a special case where compensating differentials at the current workplace and a prospective workplace are independent and uniform distributions [0,a], and disregard the expected wages and the limitation on available options. In such cases, the job mobility rate with no information is $\left(0.5a-SC\right)a$, as workers expect 0.5 compensating differentials if they have no information. With full information it is $\frac{{\left(a-SC\right)}^{2}}{2}$, i.e., is always higher than the previous term; thus, the information increases mobility. Intuitively, this is because although networks can provide good and bad news, information on one good potential workplace is enough to motivate mobility.

**Figure 3:**
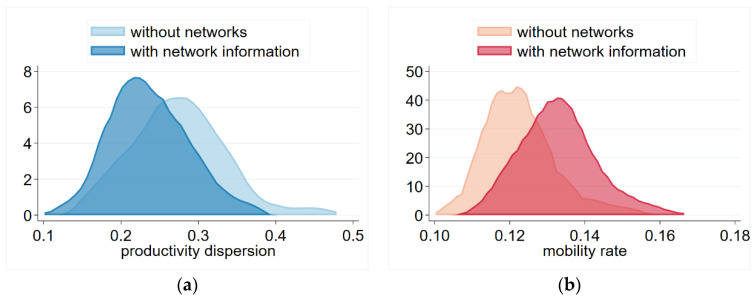
Productivity dispersion (**a**) and mobility (**b**) with and without network information. Notes: Each distribution represents 100 simulations at the 100th steps. Parameters: $N_{p}=300\;persons,\;N_{f}=30\;firms,\;\beta =0.5,\;\delta =0.1,\;\lambda =0.1,\;\theta =0.3,\;SC=0.4$.

We can also observe this phenomenon in the simulations, which showed that network information increased the chances that mobility would result (Figure 3b). It can be observed that by increasing mobility, the productivity dispersion decreased (Figure 3a). In the figure, we visualize the results of 100 simulations (at the 100th step, which is sufficient to reach the stable range after the initial adjustment based on results shown in Figure 1).

### 3.3. Comparison to Observed Mobility Patterns

To get some insights into external validity, we compared our predictions on labor mobility patterns with observational data. Glitz and Vejlin [3] reported the role of previous coworkers on the mobility of the 4.5 million workers in Denmark. They observed that 33% of mobile workers moved to firms where they had at least one former co-worker. They calculated that this figure would have been only 2.1% if mobile workers were allocated to random workplaces, and 11.4% if those were allocated to random workplaces that are filled with people with the same education, gender, and regional location as the given worker. It could be calculated that these region–gender–education cells contained on average 2760 workers (presumably with significant variance).

**Figure 4:**
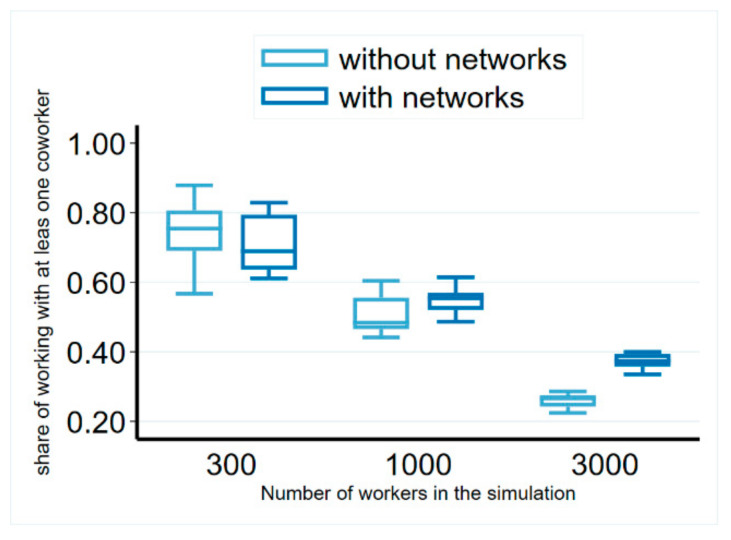
Probability of workers following each other across workplaces. Notes: Results from 10 simulations at the 100th step. Parameters: $N_{f}=30,\;100\;and\;300\;firms,\;\beta =0.5,\;\delta =0.1\;,\;\lambda =0.1,\;0.03,\;and\;0.01\;respectively\;\theta =0.3,\;SC=0.4$.

We obtained the ratio of mobile workers who moved to a workplace with at least one former co-worker being present for different sizes of labor markets (Figure 4) from the simulations. By increasing the size of the labor market, this figure decreased, due to the decreasing probability of ending up at the same place just by chance. We found that this figure was on average 37% in the network information scenario on a labor market of 3000 workers, close to the observed figure from Denmark. We also found that this was significantly decreased to 26% if no network information was available (Figure 4). However, this figure was still significantly higher than that observed in the real data on random allocation. A possible explanation for this divergence may be due to the wage differences between firms, as in the simulation, people moved to better-paying firms with a higher probability; therefore, they tended to end up at these firms with a higher probability even in the scenario without network information, whereas Glitz and Vejlin used random allocation as a comparison. In addition, firm size distributions also influence these figures, which may be different in the comparisons.

A further consideration on validity concerns the skill-level of workers. Most of the studies on knowledge transfer by labor mobility consider the mobility of inventors, e.g., [53], or R&D personnel [4], and even the more general studies we used as references observed that productivity spillovers increased by skill-level [6], or that they were only observed for highly educated workers or in professional or technical jobs [9]. Therefore, we should carefully interpret our prediction, which is relevant to the mobility of skilled personnel.

### 3.4. Regional Analysis

Our key question was whether labor mobility contributes to the convergence of the regions, and if so, how networks contribute to this. Therefore, we analyzed regions with different initial productivity levels. In the simulations we examined scenarios with significant (40%) initial average productivity differences between them.

**Figure 5:**
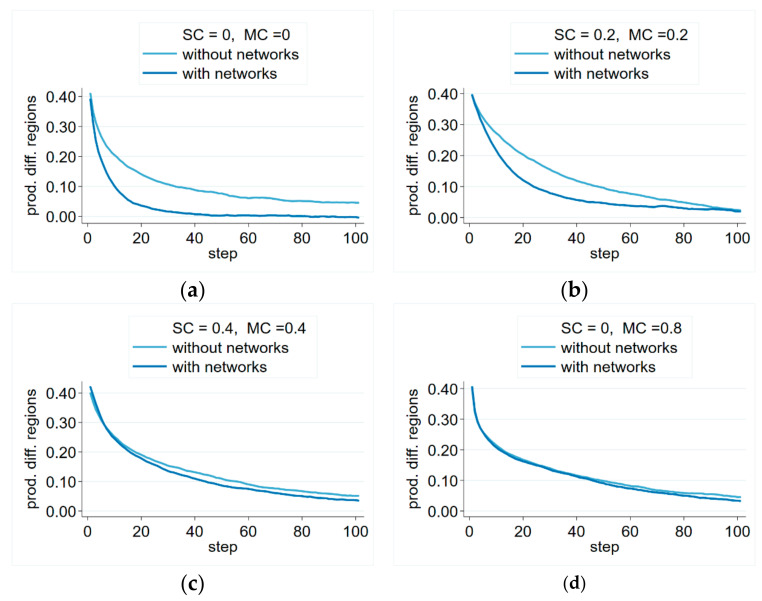
Productivity differences between regions with and without network information over different switching costs and moving cost parameters. Notes: Averages over 100 simulations. Parameters: $N_{p}=300\;persons,\;N_{f}=30\;firms,\;\beta =0.5,\;\delta =0.1,\;\lambda =0.1,\;\theta =0.3$.

Simulations indicate rapidly decreasing productivity differences between regions, and in our simulations, they reached an economically insignificant level. It could also be observed that balancing of the average productivity level was quicker in the network information scenario (Figure 5), but the effect of network information depended on the level of moving costs. If they were zero (Figure 5a), or they were moderate (Figure 5b), regional differences leveled up much more quickly if network information was present, compared to the no-network scenario. In cases where high moving costs were coupled with significant switching costs (Figure 5c), or if they were very high (Figure 5d), the advantage of network information was negligible. Nevertheless, we did not see even one example of network information sustaining regional differences—even when moving costs were very high.

**Figure 6:**
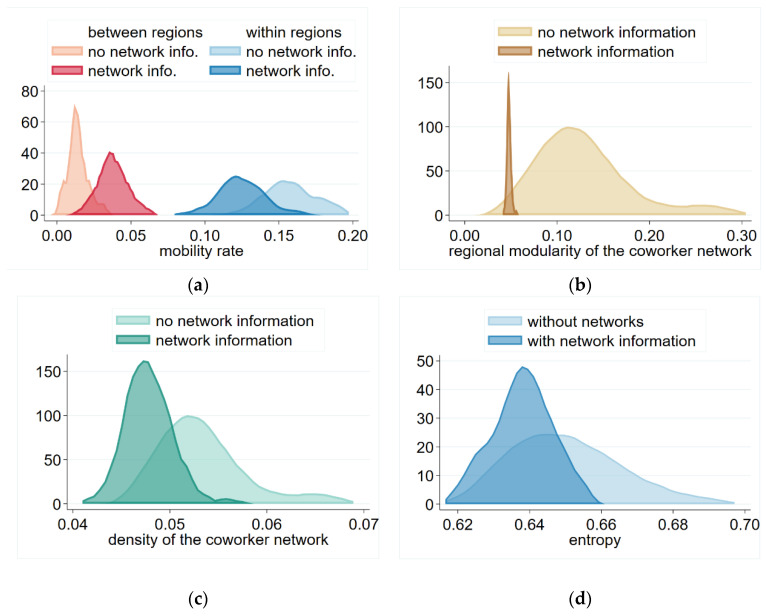
Characteristics of regional labor mobility and co-worker networks with and without network information. (**a**) Mobility between and within the regions. (**b**) Regional modularity of the coworker networks. (**c**) Density of the coworker networks. (**d**) Entropy of the co-worker networks. Notes: Each distribution represents 100 simulations at the 100th step. Parameters: $N_{p}=300\;persons,\;N_{f}=30\;firms,\;\beta =0.5,\;\delta =0.1,\;\lambda =0.1,\;\theta =0.3$.

We have seen that network information increased mobility, and this remained true in our two-region model as well. However, our regional analysis revealed an interesting feature—that network information decreased labor mobility within the region, but increased it between them (Figure 6a). These tendencies were also reflected in the network structure of the co-worker networks. The modularity of the co-worker network by region indicated the tendency to which these networks concentrated according to regions. The modularity value of a network in general is:

(5) $$mod\left(\ell \right)=\overset{K}{\underset{k=1}{\sum }}{\left[f_{kk}\left(\ell \right)-f_{kk}^{*}\right]}^{2}$$

where ℓ is a partition of the network to K modules, $f_{kk}\left(\ell \right)$ is the fraction of links within the *k-*th module compared to the whole network, and $f_{kk}^{*}$ is the same fraction under random assignment of vertices to modules. In this case, we had *K = 2* regions, and $f_{11}$ and $f_{22}$ were the fraction of links within them.

Figure 6b points out that this tendency was weaker in the network information scenario, corresponding to the higher rate of interregional mobility. In contrast, the overall density of the network (Figure 6c) was decreased in the network information scenario despite the overall higher mobility rate. To describe how much the co-worker networks follow random tendency, we calculate the network entropy measure:

(6) $$H=\frac{1}{N\ln\left(N-1\right)}\overset{N}{\underset{i=1}{\sum }}\ln k_{i}$$

where *N* is the number of nodes (workers), and $k_{i}$ is the degree of the nodes.

Entropy of the networks (Figure 6d) suggests that in the no-information scenario, movement of the workers is more random, while with network information they are more likely to follow each other across firms; thus, they expand their co-worker networks less during their career. This manifests in their lower average degree, and the subsequent lower density of the co-worker network. Comparing the density and the entropy figures, the impact of co-worker information appears to be similar on these two measures—suggesting that the decrease in entropy is due to the decreased average degree of mobility, and distribution of of mobility across workers did not became more equal.

## 4. Conclusions

Previous studies have shown empirical evidence for labor mobility resulting in knowledge-transfer observed in the productivity of firms [6,8]. Our results extend the literature in economic geography on the role of coworker networks in regional development. On the regional level, it was also observed that dense co-worker networks were associated with regional growth [15,16,17] and that those distant ties in co-worker networks provided access to new skills for firms [37]. It was also observed that labor mobility from developed countries to less developed regions induced by external shocks contributed to the development of local industries [18,19]. Our model provides a missing piece between these findings, suggesting that the micro-mechanism of voluntary labor mobility of workers is sufficient for the catching-up of less developed regions—even without external shocks. This implies that easing labor mobility between regions may have a positive impact even for less developed (peripheral) regions (or countries), as they can exploit the knowledge transfer from (return) migration.

In addition, our findings suggest that job information transmitted by social networks can amplify job-related migration, instead of constraining it. This aspect of the model connects to the literature in labor economics concerning the role of social networks on labor mobility—especially on studies focusing on how co-worker networks shape labor mobility patterns [29,32]. Our model also extends the existing knowledge on economic models of migration [54,55,56] with the inclusion of firm-level heterogeneity and dynamics to the model. However, an important difference is in contrast to, e.g., [56], is that in our model, social networks do not directly decrease mobility costs. Extension of the model with variation of switching costs based on network connections or geographical proximity would open up the new research direction of analyzing the role of labor mobility, networks, and knowledge transfer in a migration system of more than two interconnected regions with different levels of development, proximity, and network connectedness.

This model also provides new non-trivial insights into how micro-mechanisms of labor mobility influence the structure of coworker networks, and on the understanding of the empirical consequences of these. It shows that one feature of co-worker networks is that they provide information about the potential fit of workers to jobs, which induces the tendency of workers to follow each other across firms [32], which is manifested by the decreased density of co-worker networks. Moreover, the model suggests that even in the presence of limited interregional mobility (movement costs), such network information is associated with higher interregional mobility and, subsequently, decreased regional differences. However, we found that the high cost of mobility limits the effect of network information on speeding up regional convergence.

These features may promote further empirical analysis of the consequences of different co-worker network structures. In particular, it suggests that although higher density and entropy of co-worker networks could be associated with a higher potential of facilitating knowledge flows across firms, more “structured” co-worker networks with lower density and entropy can be a consequence of and indicator that these networks actually provide information about jobs, which is then utilized in labor mobility.

Concerning the modelling assumptions, we made the important choice of assuming workers to be homogenous. When modelling network information, we chose a corresponding model in which networks provide information about the employer–employee fit. However, the alternative approach is apparent, in which workers are heterogeneous and networks provide information about their “quality”, following the model of Montgomery [26]. This opens up a different research direction on the impact of networks on the selection of highly skilled workers in developed regions, or big cities, which is observed in labor economics [57,58], and has serious consequences on the development of urban–rural inequalities [59].

## Data Availability

All data in this article was generated by the agent-based simulation program, which is available within Supplementary Materials.

## Supplementary Materials

All data in this article was generated by the agent-based simulation program, which is available online at www.mdpi.com/article/10.3390/e23111451/s1.
